# The Monocyte-to-Lymphocyte Ratio at Hospital Admission Is a Novel Predictor for Acute Traumatic Intraparenchymal Hemorrhage Expansion after Cerebral Contusion

**DOI:** 10.1155/2020/5483981

**Published:** 2020-12-28

**Authors:** Jiangtao Sheng, Tian Li, Dongzhou Zhuang, Shirong Cai, Jinhua Yang, Faxiu Ding, Xiaoxuan Chen, Fei Tian, Mindong Huang, Lianjie Li, Kangsheng Li, Weiqiang Chen

**Affiliations:** ^1^Department of Microbiology and Immunology & Key Immunopathology Laboratory of Guangdong Province, Shantou University Medical College, Shantou, Guangdong, China; ^2^Department of Neurosurgery, First Affiliated Hospital of Shantou University Medical College, Shantou, Guangdong, China; ^3^Department of Neurosurgery, Second Affiliated Hospital of Shantou University Medical College, Shantou, Guangdong, China; ^4^Department of Neurosurgery, Affiliated Jieyang Hospital of Sun Yat-sen University, Jieyang, Guangdong, China; ^5^Department of Neurosurgery, Fuzhou General Hospital of Xiamen University Medical College, Fuzhou, Fujian, China

## Abstract

**Purpose:**

To explore the potential of monocyte-to-lymphocyte ratio (MLR) at hospital admission for predicting acute traumatic intraparenchymal hematoma (tICH) expansion in patients with cerebral contusion. *Patients and Methods*. This multicenter, observational study included patients with available at-hospital admission (baseline) and follow-up computed tomography for volumetric analysis (retrospective development cohort: 1146 patients; prospective validation cohort: 207 patients). Semiautomated software assessed tICH expansion (defined as ≥33% or 5 mL absolute growth). MLR was acquired from routine blood tests upon admission. We constructed two predictive models: basic combined model of clinical and imaging variables and MLR combined model of both MLR and other variables in the basic model. Receiver operating characteristic (ROC) analysis and decision curve analysis (DCA) were used to estimate the performance of MLR for predicting acute tICH expansion.

**Results:**

MLR was significantly larger in patients with acute tICH expansion compared to those without acute tICH expansion (mean [SD], 1.08 [1.05] vs. 0.59 [0.37], *P* < 0.001). A nonlinear positive relationship between MLR and the incidence of acute tICH expansion was observed. Multivariate logistic regression indicated MLR as an independent risk factor for acute tICH expansion (odds ratio (OR), 5.88; 95% confidence interval (CI), 4.02-8.61). The power of the multivariate model for predicting acute tICH expansion was substantially improved with the inclusion of MLR (AUC 0.86 vs. AUC 0.74, *P* < 0.001), as was also observed in an external validation cohort (AUC 0.83 vs. AUC 0.71, *P* < 0.001). The net benefit of MLR model was higher between threshold probabilities of 20-100% in DCA. For clinical application, a nomogram derived from the multivariate model with MLR was introduced. In addition, MLR was positively associated with 6-month unfavorable outcome.

**Conclusion:**

MLR is a novel predictor for traumatic parenchymatous hematoma expansion. A nomogram derived from the MLR model may provide an easy-to-use tool for predicting acute tICH expansion and promoting the individualized treatment of patients with hemorrhagic cerebral contusion. MLR is associated with long-term outcome after cerebral contusion.

## 1. Introduction

Hemorrhagic cerebral contusion is one of the most severe types of traumatic brain injury (TBI) that occurs in 20–30% in TBI patients [1]. It is characterized by acute traumatic intraparenchymal hematoma (tICH), leading to a high incidence of mortality and residual disability among survivors. Outcomes in patients are influenced by the presence of acute tICH expansion, which occurs in 38–63% of patients [2]. Acute tICH expansion is also an important target during early intervention after cerebral contusion. Despite extensive study and improvements in critical care, acute tICH expansion continues to be difficult to predict.

Neuroinflammation is a primary feature of tICH after cerebral contusion [3]. Evidence suggests that neuroinflammation contributes to the tICH pathology and influences its course [4, 5]. Infiltration of inflammatory cells to the brain is largely restricted by the blood-brain barrier (BBB) under normal physiological condition; however, in patients with cerebral contusions, the invasion of monocytes, neutrophils, and lymphocytes from the periphery occurs and contributes to the course of damage. Monocytes play a key role in secondary brain injury after tICH. Increased monocytes following cerebral contusion have been demonstrated to contribute to the infiltration of neutrophils in the brain which is associated with unfavorable outcomes [6, 7]. Although the role of lymphocytes in the acute phase of traumatic intraparenchymal hemorrhage is unclear, lower lymphocyte levels have been shown to be associated with spontaneous intraparenchymal hematoma expansion and clinical deterioration [8, 9].

The monocyte-lymphocyte ratio (MLR) reflects the balance of change between innate and adaptive immunity and offers a simple indicator of immune status and inflammation level. As an inexpensive and readily available biomarker, MLR recently has been suggested to predict prognosis in various diseases, including cancer [10–12], cardiovascular disease [13, 14], and neurological disorders [15, 16]. The predictive value of MLR for the acute progression of traumatic intraparenchymal hemorrhage has not been explored. In this multicenter-retrospective cohort study, the aim was to explore its predictive value for acute tICH expansion.

## 2. Patients and Methods

### 2.1. Ethics

The current study has been approved by the ethics committees of the First Affiliated Hospital of Shantou University Medical College, the Second Affiliated Hospital of Shantou University Medical College, the Affiliated Jieyang Hospital of Sun Yat-sen University, and Fuzhou General Hospital of Xiamen University and has therefore been performed in accordance with the ethical standards laid down in an appropriate version of the Declaration of Helsinki (as revised in Brazil 2013). All methods were performed in accordance with the relevant guidelines and regulations. Informed consent for study inclusion was obtained from all patients (or their surrogates) before study participation.

### 2.2. Patient Population

**Figure 1:**
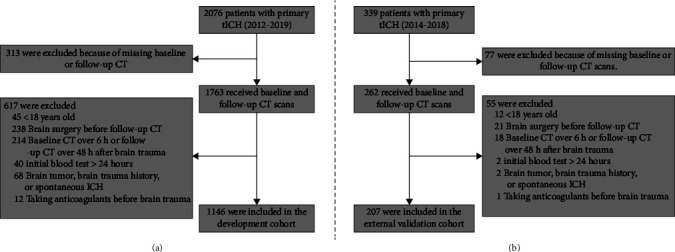
Flowchart of patient selection process including inclusion and exclusion criteria. Patients in the final cohort were retrospectively selected from the First Affiliated Hospital of Shantou University Medical College, the Second Affiliated Hospital of Shantou University Medical College, and the Affiliated Jieyang Hospital of Sun Yat-sen University between January 1, 2012, and April 30, 2019 (a). Patients in the validation cohort were prospectively selected from Fuzhou General Hospital of Xiamen University between March 2014 and June 2018 (b). CT = noncontrast computed tomography.

Consecutive patients with primary cerebral contusion who were admitted to one out of the four hospitals between January 1, 2012, and April 30, 2019, were enrolled in this retrospective cohort (Figure 1(a)). The patients in the external validation cohort shared the same inclusion criteria and were retrospectively included from Fuzhou General Hospital of Xiamen University between March 2014 and June 2018 (Figure 1(b)). All patients were treated in accordance with the standardized institutional protocol of each hospital during the recruitment period. tICH was confirmed by CT scan at baseline showing intraparenchymal hematoma. Patients were excluded from the study if the baseline CT was performed >6 h or the follow-up CT >48 h after cerebral contusion, if they had undergone surgical evacuation of the hematoma before the follow-up CT, if they had a brain tumor or a history of brain trauma or spontaneous ICH, if they had no initial blood test within 24 hours after cerebral contusion, or if they take anticoagulants before brain trauma.

### 2.3. Clinical Data

Demographic and clinical data were collected (Table 1). Injury mechanisms were divided a priori into 3 groups: severe (motor vehicle crash with patient ejection, death of another passenger, or rollover; pedestrian or bicyclist struck by a motorized vehicle; falls of more than 3 m for adults; or head struck by a high-impact object), mild (ground-level falls or running into stationary objects), and moderate (any other mechanism), as previously described [17]. Blood samples were routinely collected for full blood count analysis immediately after hospital admission. Venous blood samples were drawn by venous puncture at hospital admission for routine blood tests, including leukocyte count (reference range, 3.5–9.5 × 10^9^ cells/L), neutrophil count (reference range, 1.8–6.4 × 10^9^ cells/L), lymphocyte count (reference range, 1.1–3.2 × 10^9^ cells/L), and mononuclear cell count (reference range, 0.1–0.6 × 10^9^ cells/L). The MLR was calculated as the ratio between the absolute monocyte and lymphocyte count. Patients were identified as having a coagulation disorder if the activated partial thromboplastin time was ≥36 s, the international normalized ratio was >1.2, or the platelet count was <120 × 10^9^ platelets/L at admission [18].

### 2.4. Imaging Data

Axial noncontrast CT images were obtained at each participant's institution using standardized local protocols. Baseline CT images (5 mm slice thickness) were reviewed by three readers (J.Y., D.Z., and S.C.) who were blinded to other clinical data. Baseline and follow-up hematoma volumes were calculated from the CT images using semiautomated computer-assisted volumetric analysis (General Electric Company, Waukesha, WI, USA) [19]. Briefly, the region of interest was manually selected and automatically separated from the environment via software based on a fixed threshold in Hounsfield units (HU). The isolated regions were visually inspected and manually adjusted to ensure that the hemorrhage was visible in all three projections. Adjacent voxels were automatically summarized, which provided the hematoma volume, by using a threshold value (a fixed window of 110 and 50 HU) for distinguishing hematomas from the surrounding. When multiple intraparenchymal hematomas (ICHs) were present in the contusion region, the total volume was calculated. Acute tICH expansion was defined as a relative growth of ≥33% or absolute hematoma growth of ≥5 mL from the initial CT, as previously described [20, 21].

### 2.5. Statistical Analysis

Means (standard deviations) or medians (interquartile ranges) were used to present continuous variables. Counts (percentages) were used to present categorical variables. The demographic, clinical, and imaging characteristics and inflammatory variables were compared between patients with and without tICH expansion, as well as in patients with and without in-hospital death using chi-squared test, Fisher's exact test, *t*-test, and Mann–Whitney *U* test, as appropriate.

A logistic regression analysis was used to examine the association between MLR and acute tICH expansion and the association between MLR and 6-month unfavorable outcome. Subgroup analyses and interaction tests were used to further assess potential heterogeneity of MLR on acute tICH expansion. Subgroups included sex (male versus female), age (younger than 65 years versus no less than 65 years), GCS (mild scores of 13–15 versus moderate scores of 9–12 versus severe GCS scores of 3–8), coagulation (normal function versus coagulopathy after brain contusion), time (less than 3 versus more than 3 h from onset of brain trauma to baseline CT scan), and baseline tICH volume (less than 20 mL versus no less than 20 mL).

The logistic regression analysis was also used to select potential risk factors for acute tICH expansion. For the univariate regression analyses (Supplementary Table 1), variables with *P* < 0.20 were included in the multivariate model [22]. Independent risk factors of acute tICH expansion with forward selection procedure, retaining variables with *P* < 0.20, were selected for multivariate regression analysis.

In addition, we applied a two-piecewise linear regression model to examine the relationship between MLR and acute tICH expansion by using a smoothing function, as previously reported [23]. The threshold level (i.e., turning point, MLR = 0.89) was determined through trial and error, including the selection of turning points along a predefined interval and then choosing the turning point that provided the maximum likelihood model. We also conducted a log likelihood ratio test by comparing the one-line linear regression model with a two-piecewise linear model.

Based on the multivariate logistic regression, two models for predicting acute tICH expansion were developed: a basic model that included independent risk factors for acute tICH expansion, including coagulopathy, subdural hemorrhage, time from the onset of brain trauma to baseline CT scan (time to baseline CT), baseline tICH volume, and location of contusion, and an MLR model, which also included MLR results. The discriminative ability of the predictive models using area under the receiver operating characteristic curve was assessed [24]. Decision curve analysis (DCA) was used to determine the clinical usefulness of the models [25]. DCA provides insight into the range of predicted risks for which model has a higher net benefit than simply either classifying all patients as having the outcome or no patients as having the outcome [26]. DCA can also be used to compare the net benefit of models.

Finally, based on the MLR model, a nomogram was constructed to generate acute tICH expansion after primary cerebral contusion.

All tests of significance were two-tailed. All analyses were performed using R statistical software and associated packages (R Foundation for Statistical Computing) [27–29] and SPSS version 22 (IMB Corp., Armonk, NY, USA). This report was prepared following the Transparent Reporting of a multivariable prediction model for Individual Prognosis Or Diagnosis (TRIPOD) [30].

## 3. Results

### 3.1. Patient Characteristics

A total of 1146 patients with cerebral contusions were included into the development cohort (Figure 1(a) and Table 1). The mean monocyte count, lymphocyte count, and MLR were 0.85∗10^9^ cells/L, 1.31∗10^9^ cells/L, and 0.83, respectively (Table 1). Among these patients, 559 (44.78%) had significant acute tICH expansion.

Patients with acute tICH expansion compared to patients without acute tICH expansion had a higher leukocyte count, monocyte count and MLR and a lower lymphocyte count. Patients with tICH expansion were more likely to have a severe or moderate GCS score at admission, a higher mean arterial pressure, and a coagulation disorder. Patients with acute tICH expansion were more likely to have less time to baseline CT scan, less time from baseline CT to follow-up CT, larger baseline tICH volume, and a higher frequency of SAH and SDH (Table 1).

### 3.2. MLR as a Risk Factor for tICH Expansion

**Figure 2:**
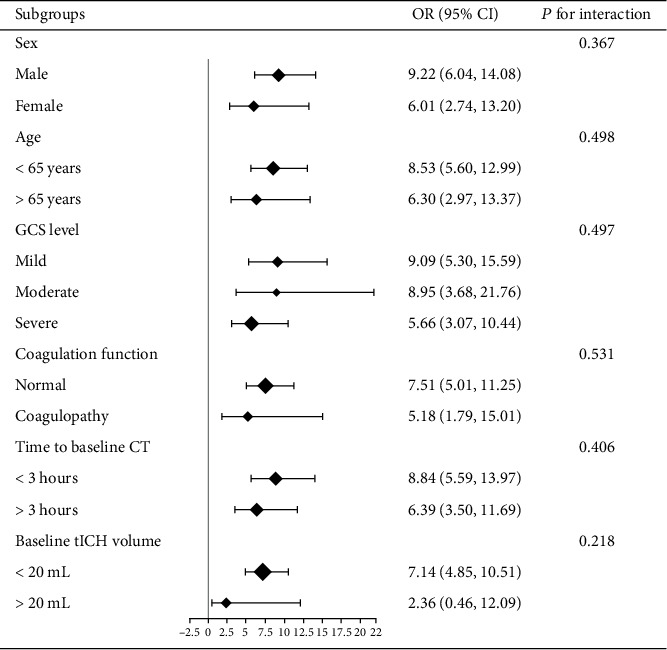
OR (95% CI) for acute tICH expansion in subgroups of cerebral contusion patients and interaction test of the stratification variables and MLR.

MLR was associated with tICH expansion (odds ratio (OR), 8.14; 95% confidence interval (CI), 5.64-11.75) based on univariate logistic regression (Table 2). Subgroup analysis indicated that the association between MLR and tICH expansion occurred across a wide spectrum of patients with cerebral contusion (Figure 2). No significant interaction was observed between MLR and any of the grouping variables, including sex, age, GCS level, coagulation function, time to baseline CT, and baseline tICH volume. The multivariate logistic regression analysis revealed that MLR remained significant after being adjusted for other independent risk factors for tICH expansion (OR, 5.88; 95% CI, 4.02-8.61) (Table 2).

**Figure 3:**
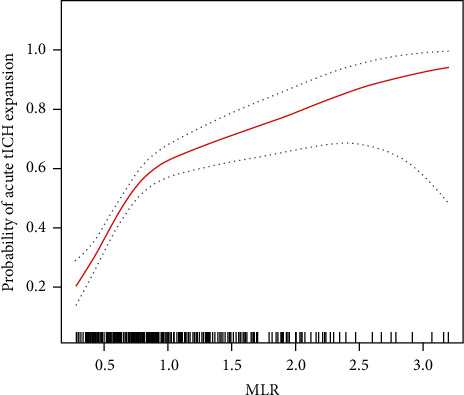
The relationship between MLR and acute tICH expansion^a^. ^a^Adjusted for sex, age, coagulopathy, subdural hemorrhage, time to baseline CT time, baseline tICH volume, and location of contusion.

Smoothed plots suggest a nonlinear relationship between MLR and the incidence of acute tICH expansion (Figure 3). The incidence of acute tICH expansion sharply increased with MLR (up to 0.89). The adjusted odds ratio (OR) of acute tICH expansion was 19.96 (95% CI, 8.08-49.32) (Table 2). However, the incidence of acute tICH expansion mildly increased when MLR was larger than 0.89, and the adjusted OR of acute tICH expansion was 2.71 (95% CI, 1.33-5.53).

### 3.3. Predictive Model with MLR for Acute tICH Expansion

**Figure 4:**
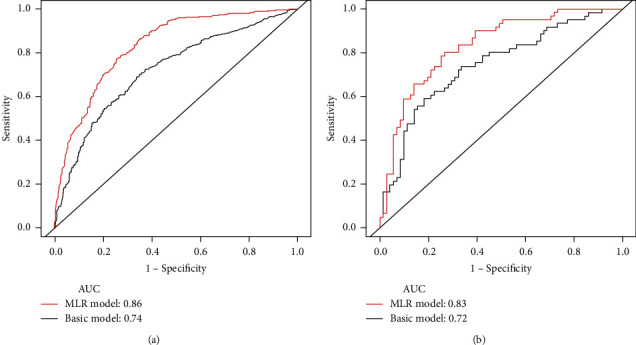
Receiver operating characteristic (ROC) curve analysis for a basic model (without MLR) and MLR model (with MLR). Black line represents the reduced model; red line represents the MLR model in the (a) development and (b) validation cohorts.

To evaluate the predictive performance of MLR, two multivariate predictive models (basic and MLR models without and with MLR, respectively) were introduced (Supplementary Table 2). Based on the results of multivariate logistic regression, subdural hemorrhage, time to baseline CT, baseline tICH volume, and location of contusion were included into the basic model. In addition to these clinical and imaging variables, the MLR model also included MLR. The area under the curve (AUC) of the MLR model was significantly larger than that of the basic model (AUC, 0.86 [95% CI, 0.80-0.92] vs. AUC, 0.74 [95% CI, 0.66-0.81]) (Figure 4(a)). The MLR model had better sensitivity than the basic model (84.06% vs. 57.97%), while specificity for acute tICH expansion was similar (77.78% vs. 79.01%).

Due to the small baseline tICH volume (mean: 4.34 mL), the predictive value of the MLR model in patients with tICH more than 20 mL was further examined. The AUC of the MLR model for acute tICH expansion was 0.86 (95% CI, 0.66-1.00). Although 55.75% (635/1146) of the patients had mild GCS scores, the MLR model performed well in patients with moderate or severe GCS scores (AUC, 0.85 [95% CI, 0.81-0.89]).

**Figure 5:**
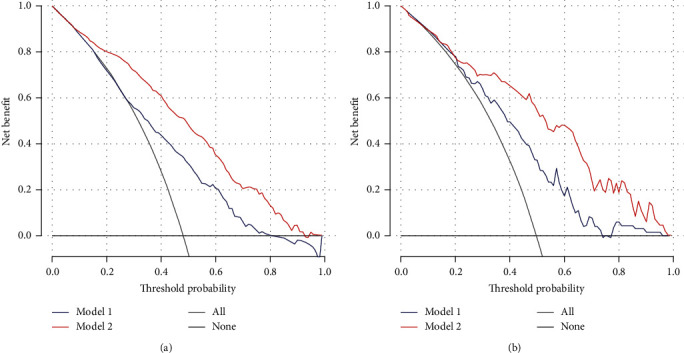
Decision curve analysis of the two models for predicting acute tICH expansion in the (a) development cohort and the (b) validation cohort, respectively. For the two predictive models, the net benefit curve is shown. Blue line (model 1) = basic model; red line (model 2) = MLR model; black line = net benefit when all the patients are considered as not having acute tICH expansion; light grey line = net benefit when all the patients are considered as having acute tICH expansion. The preferred model is the model with the highest net benefit at any given threshold.

By decision curve analysis, both models were useful between threshold probabilities of 10 and 80%, and the MLR model exhibited a better net benefit than the basic model between threshold probabilities 10 and 100% (Figure 5(a)).

### 3.4. External Validation

At Fuzhou General Hospital of Xiamen University, 207 of 339 patients with cerebral contusion were enrolled into an external validation cohort (Figure 1(b)). Acute tICH expansion occurred in 101 of 207 (48.79%) patients with primary cerebral contusion (Table 3). Patients with acute tICH expansion compared to patients without acute tICH expansion had a larger MLR (0.95 vs. 0.62, *P* < 0.001). Compared with the basic model, the MLR model exhibited a better discriminative ability for acute tICH expansion (AUC 0.83 [95% CI, 0.80-0.85] vs. AUC 0.72 [95% CI, 0.68-0.75]), with a sensitivity of 82.93% and a specificity of 69.02% (Figure 4(b)). Compared with the basic model, decision curves analysis also indicated higher net benefit of the MLR model between threshold probabilities of 20 and 100% (Figure 5(b)).

**Figure 6:**
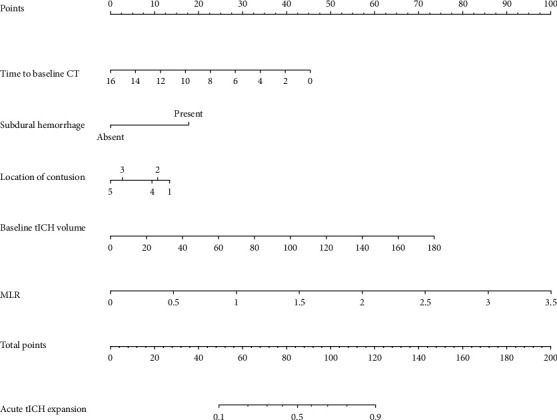
The nomogram derived from the MLR model to predict acute tICH expansion. To calculate a patient's acute tICH expansion probability, points for each parameter are assigned by corresponding values from the “points” axis, and sum of the points is plotted on “total points” axis. The patient's acute tICH expansion probability is the value at a vertical line from the corresponding total points. In the “points” axis of location of contusion, “1” represents a frontal lobe contusion; “2” represents a temporal contusion; “3” represents a parietal contusion; “4” represents an occipital contusion temporal contusion; “5” represents other location of contusion.

For clinical application, a nomogram derived from the MLR model was introduced (Figure 6). In the nomogram, each predictor was assigned a point, and the total points were acquired from a linear combination of the points of each predictor on a scale from 10% to 90% in order to evaluate the corresponding risk of acute tICH expansion.

### 3.5. Relationship between MLR and 6-Month Outcome

**Figure 7:**
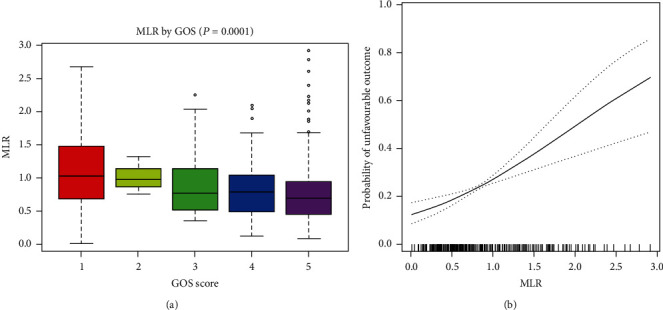
MLR was positively associated with 6-month unfavorable outcome. (a) MLR in each GOS group. (b) Relationship between MLR and the incidence of 6-month unfavorable outcome after cerebral contusion^b^. ^b^Adjusted for sex, age, coagulopathy, subdural hemorrhage, time to baseline CT time, baseline traumatic intracerebral hematoma volume, and location of contusion.

Finally, we further analyzed that association between MLR and the 6-month outcome after cerebral contusion. In the total cohort, only 37.61% (431 patients) had data for 6-month Glasgow Outcome Score (GOS). The mean MLR was 0.86 (SD, 0.53). Among them, 24.59% (106 patients) had an unfavorable 6-month outcome (GOS ≤ 3). Patients with an unfavorable outcome had higher MLR (0.79 [SD, 0.48] versus 1.07 [SD, 0.60], *P* < 0.001) than patients with a favorable outcome. As shown in Figure 7(a), patients with lower GOS typically had higher MLR. Multivariable analysis indicated that MLR was positively associated with 6-month unfavorable outcome (OR, 2.35; 95% CI, 1.02-5.13). Smoothed plots suggest that MLR were positively associated with the incidence of 6-month unfavorable outcome (Figure 7(b)).

## 4. Discussion

In this multicenter retrospective study, evidence has demonstrated that the common hematologic index ratio of the monocyte-to-lymphocyte (MLR) is strongly associated with the acute tICH expansion in the cerebral contusion after adjusting other independent clinical and imaging risk factors. The subgroup analysis indicates that this association occurs across a wide spectrum of patients with cerebral contusion. A nonlinear positive correlation between the MLR and the acute tICH expansion is also observed. The discriminative ability and the net benefit of the multivariate model for predicting the acute tICH expansion has a substantial improvement when the MLR is included in the analysis. A nomogram derived from the MLR model provides an easy-to-use tool for predicting the tICH expansion. In addition, the high MLR is positively associated with 6-month unfavorable outcome.

The MLR is used to predict the prognosis in various diseases, including cancer and cardiovascular disease [10–14]. In central nervous system (CNS) diseases, the MLR is used to predict the prognosis of the acute ischemic stroke and the spontaneous ICH [31, 32]. Additionally, the MLR is independently associated with the hemorrhagic transformation in patients with acute ischemic stroke and neurological disability and the brain atrophy in patients with multiple sclerosis [15, 16]. The relationship between the MLR and the acute progression of the hemorrhagic cerebral contusion remains unclear. To our knowledge, the current study is the first to report an association with the MLR and the acute progression after the traumatic intraparenchymal hemorrhage, which suggests the predictive value of the MLR for the acute traumatic parenchymal hematoma expansion.

The nomogram derived from the MLR model is easy to use and only requires several readily available clinical and imaging variables at admission, which can be used to identify patients at risk of acute tICH expansion (10% to 90%). Patients at high risk of tICH expansion can benefit from the use of safe and cost-effective therapies, such as hematoma/contusion evacuation and administration of recombinant activated factor VII [33], and intensive surveillance. Patients at low risk for the tICH expansion may benefit from avoiding nonessential repeat CT scans and intensive surveillance. Therefore, the nomogram may help individualize treatment in this patient population. The use of the nomogram and its benefit in patients with cerebral contusion remains to be validated in an independent cohort.

The MLR inflammatory marker reflects the activation of the innate immune system (numerator) and the repression of the adaptive immune system (denominator), offering a simple indicator for the relative balance between innate and adaptive immunity. For patients with acute cerebral contusion, the MLR provides an easy parameter to assess the individual neuroinflammatory status.

The neuroinflammation, a key feature of acute cerebral contusion associated with the tICH expansion, can influence its course and potentially represent a target for the intervention [34–36]. The neuroinflammation begins after the cerebral contusion onset. The impact injury leads to the shearing of tissue and fracturing of microvessels, resulting in initial hematoma. Danger-associated molecular patterns derived from hematoma components initiate innate immune signaling via activating astrocyte and microglia, which release multiple proinflammatory cytokine and chemokines to recruit the peripheral monocyte/macrophage and the neutrophil [35]. These peripheral white blood cells further activate multiple inflammatory pathways, like NF-*κ*B signal, which plays a crucial role in induced necrotic death of vascular endothelial cells, resulting in delayed microvessel fragmentation surrounding the initial hematoma and subsequent tICH expansion [1]. In addition, increased levels of matrix metalloproteinases (MMPs) after the ICH contribute to the delayed tICH expansion by favoring the loss of vascular integrity, thereby increasing the permeability of vascular walls [36, 37]. MMPs also promote the disruption of the blood-brain barrier and increase the infiltration of monocytes and neutrophils [38]. In turn, the development of the ICH and extravasation of leukocytes into the brain parenchyma amplify the reactions, exacerbate the cerebral injury in a vicious circle [38, 39], and deteriorate the tICH expansion and edema, thereby negatively affecting the cerebral contusion recovery.

In this study, we have observed that increased MLR is positively associated with a high incidence of 6-month unfavorable outcome. Consistently, in patients with mild and severe TBI, elevated serum levels of inflammatory cytokines, namely, CCL-2, IL-1*β*, and IL-33, are associated with unfavorable prognosis after the TBI [40, 41]. In addition, many clinical studies have reported other inflammatory indices including neutrophil-to-lymphocyte ratio and MMPs that are associated with the spontaneous ICH expansion and the clinical neurological deterioration [35, 36]. Moreover, several preclinical studies suggest that the potential therapeutic intervention with the use of the glycoprotein erythropoietin (EPO) and the glibenclamide has demonstrated neuroprotective effects through several anti-inflammatory effects in patients with cerebral contusion, but the EPO shows mixed results in recent clinical trials [42–46]. In the current clinical practice, no therapy targeting the tICH-induced primary injury has yielded conclusive benefits, and the tICH treatment remains supportive within a framework of the general critical care management. Therefore, our findings may help to identify new potential targets from neuroinflammation and develop novel therapeutic strategies.

The role of lymphocytes in the acute tICH expansion remains unclear. Consistent with previous studies, the current study shows low lymphocytes in patients with the tICH expansion, which is likely attributed to decreased T lymphocytes [47, 48]. The reduction in the T lymphocyte count is associated with the remarkable deterioration of neurologic outcomes and increased incidence of pulmonary infection in patients with traumatic brain injury [49]. Considering the presence of multiple subtypes and bidirectional immunomodulatory roles, the potential role of T lymphocytes in the acute tICH expansion may be complicated. Considering the proinflammatory environment in the early stages of cerebral contusion [50, 51], we have speculated that anti-inflammatory T lymphocyte subtypes (e.g., regulatory T (Treg) and Th2) may be depleted, thereby promoting a proinflammatory state of the immune system. However, further clinical studies designed to explore the role of T lymphocytes in the acute tICH expansion and long-term outcome after cerebral contusion are still needed.

The strengths of the current study include the multicenter design and large sample size, the externally validated and well-characterized sample of patients with cerebral contusions, and quantified measures of tICH volume, imaging pathology, and clinical variables. The datasets allow for robust statistical modeling. In addition, the discrimination of the multivariate model for predicting the acute tICH expansion is substantially improved after including the MLR. Finally, the clinical and the imaging variables needed to inform the nomogram derived from the MLR model are readily assessed upon hospital admission, thus providing an easy-to-use tool to support clinicians in their prognosis of the acute tICH expansion.

Several limitations should be considered when interpreting the current findings. First, the retrospective design may lead to selection and information biases. Although we have minimized the effect of selection bias through the use of the multicenter study design and externally validated predictive model, a prospective multicenter cohort study is still needed to independently validate the predictive value for the tICH expansion. Second, only the MLR on admission is calculated and used for predicting the tICH expansion. A dynamic monitoring for MLR may help provide comprehensive insights into the relationship between the MLR and the acute tICH expansion and identify an optimal timepoint of MLR for predicting the tICH expansion and the outcome. Lastly, the routine blood test only provides a gross metric of monocytes and lymphocytes and cannot identify monocyte and lymphocyte subsets, such as nonclassical monocytes and T lymphocyte that are related to the neurological deterioration after the cerebral contusion [6, 49].

## 5. Conclusions

In conclusion, this study suggests that the MLR upon hospital admission is an independent risk factor for the acute tICH expansion and the long-term unfavorable outcome. The MLR substantially improves the performance of the multivariate model for the acute tICH expansion after cerebral contusion. A nomogram derived from the model provides an easy-to-use tool for predicting the acute tICH expansion in patients with cerebral contusion and helps optimize the classification and the management of patients with cerebral contusion.

## Figures and Tables

**Table 1:** Baseline characteristics according to acute tICH expansion.

Variable		Acute tICH expansion		
	Total (*n* = 1146)	No (*n* = 587)	Yes (*n* = 559)	*P* value
*Demographics and clinical variables*				
Male sex, no. (%)	862 (75.22%)	438 (74.62%)	424 (75.85%)	0.629
Mean age (SD) (y)	47.97 (18.01)	46.94 (17.90)	49.06 (18.08)	0.046
Severity of injury mechanism, no. (%)				0.370
Mild	250 (28.31%)	129 (27.56%)	121 (29.16%)	
Moderate	41 (4.64%)	26 (5.56%)	15 (3.61%)	
Severe	592 (67.04%)	313 (66.88%)	279 (67.23%)	
Level on Glasgow Coma Scale score, no. (%)				<0.001
Mild (13–15 points)	635 (55.75%)	361 (61.92%)	274 (49.28%)	
Moderate (9–12 points)	215 (18.88%)	91 (15.61%)	124 (22.30%)	
Severe (3–8 points)	289 (25.37%)	131 (22.47%)	158 (28.42%)	
Mean arterial pressure, median (IQR) (mmHg)	98.67 (89.33-108.50)	97.67 (89.00-107.08)	99.67 (90.16-110.00)	0.029
Hypertension, no. (%)	116 (10.52%)	53 (9.38%)	63 (11.71%)	0.207
Diabetes, no. (%)	50 (4.48%)	22 (3.84%)	28 (5.17%)	0.285
Coagulopathy, no. (%)	97 (10.40%)	41 (8.65%)	56 (12.20%)	0.076
*Imaging variables*				
Time to baseline CT, median (IQR) (h)	2.33 (1.50-3.80)	2.50 (1.50-4.00)	2.00 (1.50-3.33)	<0.001
Time from baseline CT to follow-up CT (IQR) (h)	18.00 (10.00-24.00)	18.00 (11.00-24.00)	17.00 (8.18-24.00)	0.016
Intraventricular hemorrhage, no. (%)	71 (6.22%)	34 (5.80%)	37 (6.65%)	0.551
Subarachnoid hemorrhage, no. (%)	847 (74.10%)	392 (66.89%)	455 (81.69%)	<0.001
Subdural hemorrhage, no. (%)	726 (63.41%)	297 (50.68%)	429 (76.74%)	<0.001
Extradural hemorrhage, no. (%)	232 (20.30%)	118 (20.14%)	114 (20.47%)	0.890
Location of contusion, no. (%)				0.007
Frontal	498 (43.49%)	241 (41.13%)	257 (45.97%)	
Temporal	514 (44.89%)	257 (43.86%)	257 (45.97%)	
Parietal	60 (5.24%)	40 (6.83%)	20 (3.58%)	
Occipital	26 (2.27%)	16 (2.73%)	10 (1.79%)	
Basal ganglia, brainstem, or cerebellum	47 (4.10%)	32 (5.46%)	15 (2.68%)	
Baseline tICH volume, mean (SD) (mL)	4.34 (9.24)	2.86 (6.33)	5.66 (8.69)	<0.001
Follow-up tICH volume, mean (SD) (mL)	8.56 (15.49)	3.12 (9.58)	14.75 (19.60)	<0.001
*Inflammatory index parameters*				
Leukocyte count (∗10^9^ cells/L)	15.30 (5.55)	14.35 (5.49)	16.32 (5.44)	<0.001
Monocyte count (∗10^9^ cells/L)	0.85 (0.48)	0.83 (0.48)	0.88 (0.49)	0.047
Lymphocyte count (∗10^9^ cells/L)	1.31 (0.83)	1.65 (0.95)	0.94 (0.44)	<0.001
MLR	0.83 (0.81)	0.59 (0.37)	1.08 (1.05)	<0.001

**Table 2:** Associations of MLR with acute tICH expansion.

Models	Unadjusted		Adjusted^a^	
	OR (95% CI)	*P* value	OR (95% CI)	*P* value
MLR	8.14 (5.64, 11.75)	<0.001	5.88 (4.02, 8.61)	<0.001
MLR < 0.89	24.79 (12.25, 50.17)	<0.001	19.96 (8.08, 49.32)	<0.001
MLR ≥ 0.89	2.97 (1.69, 5.24)	<0.001	2.71 (1.33, 5.53)	0.006

^a^Adjustment by sex, age, coagulopathy, subdural hemorrhage, time to baseline CT time, baseline traumatic intracerebral hematoma volume, and location of contusion.

**Table 3:** Baseline characteristics according to acute tICH expansion in the validation cohort.

Variable		Acute tICH expansion		
	Total (*n* = 207)	No (*n* = 106)	Yes (*n* = 101)	*P* value
*Demographics and clinical variables*				
Male sex, no. (%)	155 (74.88%)	78 (73.58%)	77 (76.24%)	0.660
Mean age (SD) (y)	47.95 (17.63)	48.19 (17.85)	47.70 (17.48)	0.845
Severity of injury mechanism, no. (%)				0.850
Mild	45 (29.03%)	24 (30.00%)	21 (28.00%)	
Moderate	5 (3.23%)	2 (2.50%)	3 (4.00%)	
Severe	105 (67.74%)	54 (67.50%)	51 (68.00%)	
Level on Glasgow Coma Scale score, no. (%)				0.636
Mild (13–15 points)	115 (55.83%)	61 (58.10%)	54 (53.47%)	
Moderate (9–12 points)	34 (16.50%)	18 (17.14%)	16 (15.84%)	
Severe (3–8 points)	57 (27.67%)	26 (24.76%)	31 (30.69%)	
Mean arterial pressure, median (IQR) (mmHg)	98.66 (88.08-110.00)	97.00 (87.33-113.33)	99.33 (88.67-108.67)	0.645
Hypertension, no. (%)	20 (9.95%)	10 (9.90%)	10 (10.00%)	0.981
Diabetes, no. (%)	3 (1.51%)	100 (99.01%)	96 (97.96%)	0.543
Coagulopathy, no. (%)	17 (10.37%)	6 (7.06%)	11 (13.92%)	0.150
*Imaging variables*				
Time to baseline CT, median (IQR) (h)	2.00 (1.36-3.50)	2.31 (1.50-3.33)	2.00 (1.11-3.50)	0.029
Time from baseline CT to follow-up CT (IQR) (h)	16.00 (8.03-24.00)	18.00 (10.50-24.00)	11.50 (6.50-24.00)	0.058
Intraventricular hemorrhage, no. (%)	15 (7.25%)	7 (6.60%)	8 (7.92%)	0.715
Subarachnoid hemorrhage, no. (%)	161 (77.78%)	75 (70.75%)	86 (85.15%)	0.013
Subdural hemorrhage, no. (%)	137 (66.18%)	55 (51.89%)	82 (81.19%)	<0.001
Extradural hemorrhage, no. (%)	43 (20.77%)	18 (16.98%)	25 (24.75%)	0.168
Location of contusion, no. (%)				0.013
Frontal	88 (42.51%)	37 (34.91%)	51 (50.50%)	
Temporal	97 (46.86%)	52 (49.06%)	45 (44.55%)	
Parietal	8 (3.86%)	8 (7.55%)	0 (0.00%)	
Occipital	6 (2.90%)	3 (2.83%)	3 (2.97%)	
Basal ganglia, brainstem, or cerebellum	8 (3.86%)	3 (2.83%)	3 (2.97%)	
Baseline tICH volume, mean (SD) (mL)	5.31 (14.65)	2.86 (6.33)	5.66 (8.69)	<0.001
Follow-up tICH volume, mean (SD) (mL)	8.85 (15.24)	3.12 (9.58)	14.75 (19.60)	<0.001
*Inflammatory index parameters*				
Leukocyte count (∗10^9^ cells/L)	15.34 (5.37)	14.33 (5.63)	16.43 (4.89)	<0.001
Monocyte count (∗10^9^ cells/L)	0.82 (0.43)	0.79 (0.43)	0.87 (0.48)	0.051
Lymphocyte count (∗10^9^ cells/L)	1.27 (0.65)	1.56 (0.70)	0.95 (0.40)	<0.001
MLR	0.78 (0.53)	0.62 (0.46)	0.95 (0.56)	<0.001

## Data Availability

The datasets generated during and/or analyzed are available from the corresponding authors upon reasonable request.

## Supplementary Materials

Supplementary Materials Supplementary Table 1: univariable analysis of predictive factors for acute tICH expansion. Supplementary Table 2: the basic and MLR models for predicting acute tICH expansion.
