# Supplementary material for: The Monocyte-to-Lymphocyte Ratio at Hospital Admission Is a Novel Predictor for Acute Traumatic Intraparenchymal Hemorrhage Expansion after Cerebral Contusion
Source: Mediators Inflamm. 2020 Dec 28;2020:5483981. doi: 10.1155/2020/5483981 (PMC7785383; doi:10.1155/2020/5483981)
Supplement: Supplementary Materials — Supplementary Table 1: univariable analysis of predictive factors for acute tICH expansion. Supplementary Table 2: the basic and MLR models for predicting acute tICH expansion. [file 5483981.f1.zip › Supplementary Table 1.docx]

**Supplementary Table 1. Univariable analysis of predictive factors for acute tICH Expansion**

| **Variable** | **Odds Ratio (95% CI)** | ***P* Value** |
| --- | --- | --- |
| Sex | 1.09 (0.83, 1.43) | 0.535 |
| Age, y | 1.01 (1.00, 1.01) | 0.077 |
| Level on Glasgow Coma Scale score, no. (%) |  |  |
| Mild (13–15 points) | 1 [Reference] | 1 [Reference] |
| Moderate (9–12 points) | 1.80 (1.31, 2.47) | <0.001 |
| Severe (3–8 points) | 1.65 (1.24, 2.19) | <0.001 |
| Mean arterial pressure, median, mmHg | 1.01 (1.00, 1.01) | <0.051 |
| Hypertension (Yes vs No) | 1.25 (0.85, 1.85) | 0.250 |
| Diabetes (Yes vs No) | 1.34 (0.76, 2.37) | 0.318 |
| Coagulopathy (Yes vs No) | 1.37 (0.89, 2.10) | 0.253 |
| Time to baseline CT, h | 0.88 (0.83, 0.94) | <0.001 |
| Time from baseline CT to follow-up CT (IQR), h | 0.99 (0.98, 1.00) | 0.012 |
| Intraventricular hemorrhage (Yes vs No) | 1.30 (0.79, 2.12) | 0.310 |
| Subarachnoid hemorrhage (Yes vs No) | 2.27 (1.72, 3.00) | <0.001 |
| Subdural hemorrhage (Yes vs No) | 3.16 (2.45, 4.08) | <0.001 |
| Epidural hemorrhage (Yes vs No) | 1.05 (0.78, 1.41) | 0.742 |
| Location of contusion |  |  |
| Parietal | 1 [Reference] | 1 [Reference] |
| Frontal | 2.17 (1.22, 3.87) | 0.009 |
| Parietal | 2.04 (1.14, 3.64) | 0.016 |
| Occipital | 1.25 (0.48, 3.27) | 0.650 |
| Basal ganglia, brainstem, or cerebellum | 1.00 (0.44, 2.29) | 0.754 |
| Baseline tICH volume (per 10-ml increase) | 1.40 (1.20, 1.70 | <0.001 |
