# Supplementary material for: The Monocyte-to-Lymphocyte Ratio at Hospital Admission Is a Novel Predictor for Acute Traumatic Intraparenchymal Hemorrhage Expansion after Cerebral Contusion
Source: Mediators Inflamm. 2020 Dec 28;2020:5483981. doi: 10.1155/2020/5483981 (PMC7785383; doi:10.1155/2020/5483981)
Supplement: Supplementary Materials — Supplementary Table 1: univariable analysis of predictive factors for acute tICH expansion. Supplementary Table 2: the basic and MLR models for predicting acute tICH expansion. [file 5483981.f1.zip › Supplementary Table 2.docx]

**Supplementary Table 2. The** **basic and MLR models for predicting acute tICH Expansion.**

| **Variable** | **Basic model** | | **MLR model** | |
| --- | --- | --- | --- | --- |
|  | **Odds Ratio (95% CI)** | ***P* Value** | **Odds Ratio (95% CI)** | ***P* Value** |
| Subdural hemorrhage (Yes vs No) | 2.89 (2.09, 4.00) | <0.001 | 2.61 (1.81, 3.77) | <0.001 |
| Time to baseline CT, h | 0.83 (0.76, 0.90) | <0.001 | 0.84 (0.77, 0.92) | <0.001 |
| Baseline tICH volume (per 10-ml increase) | 1.30 (1.10, 1.50) | 0.009 | 1.20 (1.00, 1.40) | <0.037 |
| Location of contusion |  |  |  |  |
| Parietal | 1 [Reference] | 1 [Reference] | 1 [Reference] | 1 [Reference] |
| Frontal | 2.46 (1.20, 5.01) | 0.014 | 2.12 (0.97, 4.60) | 0.038 |
| Parietal | 2.00 (0.97, 4.00) | 0.062 | 1.72 (0.79, 3.75) | 0.170 |
| Occipital | 2.35 (0.71, 7.75) | 0.650 | 1.52 (0.42, 5.53) | 0.530 |
| Basal ganglia, brainstem, or cerebellum | 0.85 (0.30, 2.44) | 0.890 | 0.70 (0.22, 2.18) | 0.535 |
| MLR | — | — | 5.88 (4.02, 8.61) | <0.001 |
